# Rapid and Esthetic Wound Healing in Surgery by Applying a Powdered Mixture of Mannose and Calcium Propionate

**DOI:** 10.1007/s00266-025-05186-z

**Published:** 2025-09-04

**Authors:** Cristian Ionuţ Ciucanu, Sonia Raţiu, Sorin Olariu, Ionel Ciucanu

**Affiliations:** 1 Faculty of Medicine, University of Medicine and Pharmacy “Victor Babes” of Timişoara, Timişoara, Romania; 2 Department of Chemistry, West University of Timisoara, Timişoara, Romania

**Keywords:** Esthetic wound healing, Skin, Surgery, Bleeding time, Calcium propionate, D-Mannose

## Abstract

**Background:**

Wound healing is a current problem in surgery. Wounds most commonly occur from surgery, accidents, and burns. The purpose of this study was to reduce bleeding time and wound healing time as well as prevent or mitigate scarring by applying D-mannose with calcium propionate mixtures directly to the wound in powder form.

**Methods:**

This study was a placebo, randomized, double-blind, crossover experiment involving a single group of twenty-five subjects. The number of incisions made was equal to the number of treatments required. Each subject had one incision per day. Only one treatment was applied to each wound. By the end, each subject was treated with all test mixtures and the placebo substance.

**Results:**

The results show that the bleeding time can be reduced to about 50% compared to D-mannose when treating the wound with a mixture containing 20-60 moles of D-mannose per 1 mole of powdered calcium propionate. The wound healing time was reduced by about 40% compared to D-mannose. No visible scarring was observed after 40 days on skin treated with the mannose and calcium propionate mixture.

**Conclusions:**

Bleeding time can be reduced by approximately 50% when the wound is treated with a mixture containing 40 moles of D-mannose per 1 mole of calcium propionate and wound healing time by approximately 40% compared to D-mannose. Both substances contribute to the destruction of pathogens and to the processes of new tissue formation, which accelerates the healing process. Treatment with this powdered mixture prevents skin scarring.

**Level of Evidence I:**

This journal requires that authors assign a level of evidence to each article. For a full description of these Evidence-Based Medicine ratings, please refer to the Table of Contents or the online Instructions to Authors www.springer.com/00266.

## Introduction

Wound healing is a current problem in surgery. Research over the past decade has led to a substantial increase in the methods and agents used to stop bleeding and heal wounds [1, 2]. Although various wound healing agents have been developed, improvements in efficacy, toxicity, and ease of handling are constantly required.

Wounds most commonly occur from surgery, accidents, and burns and can be internal or external. In external wounds, the skin is broken and damaged, which is very dangerous, because the subcutaneous tissue will be continuously exposed to the action of external pathogens. Improper healing leads to the formation of scars or chronic wounds that are much more difficult to treat and generate very high costs.

The healing of injured tissues is a complex natural process that consists of hemostasis, the inflammatory process, tissue formation, and remodeling of damaged tissues [3].

These processes are dynamic and overlapping in time. When tissue is injured, impulses are sent from the injured site to the hypothalamus that will trigger a series of cascading changes in metabolic and physiological processes to restore homeostasis. The first changes will occur primarily to stop the bleeding, and at the same time, the innate immune system will be triggered to protect against pathogens.

Intracellular and extracellular calcium has a multitude of roles in stimulating the healing process. Calcium ions have a special role in hemostasis, as they represent coagulation factor IV and are involved in the activation of other coagulation factors. The influx of calcium ions is a stimulus in a series of cascade processes such as platelet aggregation and the conversion of fibrinogen to fibrin. A wide variety of fundamental cell functions are regulated by very fine changes in extracellular calcium ion concentration. Thus, a slight increase in the concentration of calcium ions will cause the activation of platelets [4], which are hematopoietic cells that will adhere to the ruptured wall to block blood flow. For this reason, calcium was used in the hemostasis process in the form of calcium chloride [5], calcium alginate [6], calcium fluoride [7], calcium phosphate [8], calcium–phosphate-based nanoparticles [9], and the calcium salt of rosuvastatin [10]. In addition, calcium ions have been shown to play a role in the proliferation, differentiation, and maturation of keratinocytes and fibroblasts, as well as other functions through signal transduction and gene expression [11]. In these studies, it was found that some calcium salts also possess a slight antibacterial [7] or anti-inflammatory [12] action.

D-mannose has been found to be a substance with beneficial wound healing properties [13]. Our strategy was to find a calcium salt with an acid with more pronounced antiseptic and anti-inflammatory properties, which together with D-mannose would contribute to the acceleration of wound healing. That is why we chose calcium propionate that has not been used in wound healing before but is known as a good food preservative. Propionate anion and implicitly propionic acid have proven to be very effective in destroying a large number of pathogens including multi-resistant Gram-positive and Gram-negative bacteria and fungi [14, 15]. In addition, the propionate ion has proven anti-inflammatory effects. Given these results, we attempted to combine the beneficial properties of calcium propionate with those of D-mannose to reduce healing time and wound scarring. There is no study on wound healing by applying a powdered mixture of calcium propionate and D-mannose directly to the wound surface.

The purpose of this study is to investigate the bleeding time and wound healing time obtained by direct application to the wound surface of a mixture consisting of D-mannose with calcium propionate in powder form with the aim of reducing bleeding time, wound healing time, and visibly reducing scarring.

## Materials and Methods

### Materials

The calcium propionate crystals were 99% pure, and the D-mannose powder was microbiologically pure. The calcium propionate powder was obtained by finely grinding the calcium propionate crystals with a mortar.

## Subjects

Twenty-five Caucasian men over the age of 50 participated in this study. Subjects with any form of cardiovascular disease, diabetes, kidney or liver disease, thyroid disease, autoimmune disorders, smoking, and inflammatory diseases were excluded. Subjects did not take any medication during the study period. All followed the same regular diet throughout the study. They completed a personal medical history questionnaire and a complete physical examination. In this study, we selected only volunteers who had values close to the range of healthy patients over 50 years of age for platelets, white blood cell count, total serum calcium, serum ionic calcium as well as serum sodium, potassium, and magnesium. Subjects did not receive any incentive to participate in this study.

## Study Design

This study was a placebo, randomized, double-blind, crossover experiment involving a single group of subjects. In the same areas on the lower limbs of each subject, incisions with a depth of 4 mm and a length of 5 mm were made using a surgical knife with a depth limiting device. Each area was previously cleaned of hair and was sterilized according to the surgical protocol. The incision area was locally anesthetized with 1-2 puffs of kellen spray. The incision involved the skin and subcutaneous tissue. The wounds were not sutured. Immediately after the incision, each wound was opened and the treatment procedure followed.

The number of incisions made was equal to the number of treatments required. One incision per day was made on each volunteer. Only one treatment was applied to each wound. Treatments were randomized so that at the end, each subject was treated with all powder mixtures of D-mannose and calcium propionate and D-mannose powder used as a placebo. Details about the ratio of D-mannose and calcium propionate mixtures are presented in the Results section.

All wounds were covered with sterile non-adhesive dressings. Wound dressings were immobilized with elastic adhesive bandages. During the next 7 days, this procedure was repeated every day after the wounds were previously cleaned with medical distilled water. After this period, the wounds were protected only with a sterile dressing.

## Bleeding Time Evaluation

In the bleeding time step, the blood that flowed after the application of the test substance to the wound was absorbed with a filter paper. The procedure is repeated periodically until no more blood flows. For each test, the time until blood flow stopped was measured with a stopwatch. The bleeding time for the placebo test of each volunteer was used as the reference value for the bleeding time values of the substances tested on that volunteer.

The relative bleeding time of all wounds was calculated using the following equation:

1 $${\text{Relative bleeding time }}\left( \% \right) \, = \, \left( {{\text{BTt }}/{\text{ BTp}}} \right) \, \times { 1}00,$$

where BTt and BTp represent the bleeding time for treatment and placebo substance, respectively.

## Wound Evaluation

The wound healing evaluation was photographed with a digital camera, with 8 magnifications. Wound length and width were measured daily for the first few days and then periodically and entered into the ellipse area formula, as the wound area was approximated by the area of an ellipse. The relative wound area of all wounds was calculated using the following equation:

2 $${\text{Relative wound area }}\left( \% \right) \, = \, \left( {\text{Ai/Ao}} \right) \, \times { 1}00,$$

where Ai and Ao represent the area of the wound after *i* days of healing and the area on the day the wound was made, respectively.

## Statistical Analyses

The variables measured in this study were relative bleeding time and the change over time in the relative area of wounds on each subject of the study group. The variables of the statistical analysis are expressed as mean ± standard deviation (SD). Student’s paired t-test was used for statistical analysis. Differences with P-values of < 0.05 were considered statistically significant.

## Results

Propionates and propionic acid are found in nature in significant amounts in the diet and in the human body. Calcium propionate is soluble in water and dissociates into calcium and propionate ions. Propionic acid and propionates are most frequently analyzed by chromatographic methods [16, 17].

D-Mannose is a monosaccharide present in many foods and in the human body and has not been shown to be a toxic substance for humans. D-mannose is also found in the blood in the free state and can be determined by chromatographic techniques [18].

**Fig. 1:**
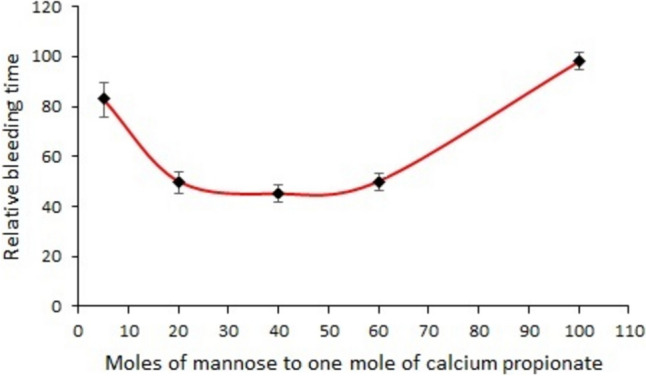
Relative bleeding time as a function of the number of moles of D-mannose per mole of calcium propionate.

Figure 1 shows the change in relative wound bleeding time as a result of increasing moles of D-mannose in a mixture with calcium propionate. The number of moles of D-mannose in the mixture was reported to one mole of calcium propionate. The relative wound bleeding time was expressed as a percentage and was obtained with Equation (1). The bleeding time when the wound was treated with the mixture of D-mannose with calcium propionate powder was compared to the bleeding time when the wound was treated with D-mannose powder alone as placebo substance. These were the pairs of observed values for statistical analysis. The P-valuse for all measurements were less than 0.05, indicating that the change was significant. It can be seen from Fig. 1 that the bleeding time can be reduced to about 50% compared to D-mannose when treating the wound with a powder mixture containing 20–60 moles of D-mannose per 1 mole of calcium propionate. Taking into account that the bleeding time is relatively constant the shortest in the range 20:1–60:1 of the molar ratio D-mannose\calcium propionate, the middle value of 40:1 was chosen in the following tests.

**Fig. 2:**
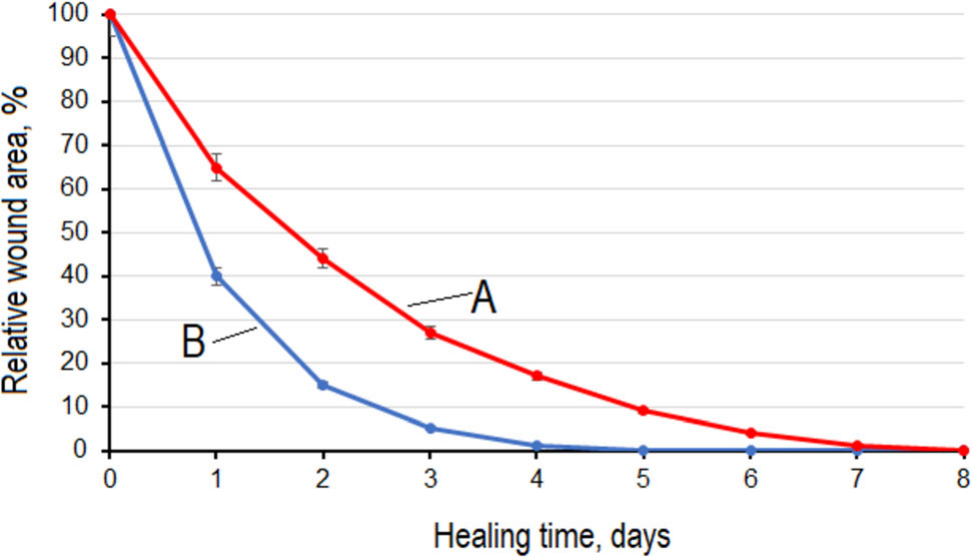
Change over time in the relative area of the wound: **A** treatment with D-mannose powder and **B** treatment with a powder mixture consisting of D-mannose and calcium propionate in a ratio of 40:1.

Figure 2 shows the change over time in the relative area of the wound for two different treatments. Curve A illustrates treatment with D-mannose powder and curve B illustrates treatment with a powder mixture consisting of D-mannose and calcium propionate in a ratio of 40:1. The wound area before treatment was compared with the wound area after treatment. The P-values for all measures before treatment and after treatment were less than 0.05, indicating that the change was significant. It can be seen that the wound healing time by treatment with the powder mixture, consisting of D-mannose and calcium propionate in a ratio of 40:1, was reduced by about 40% compared to D-mannose.

**Fig. 3:**
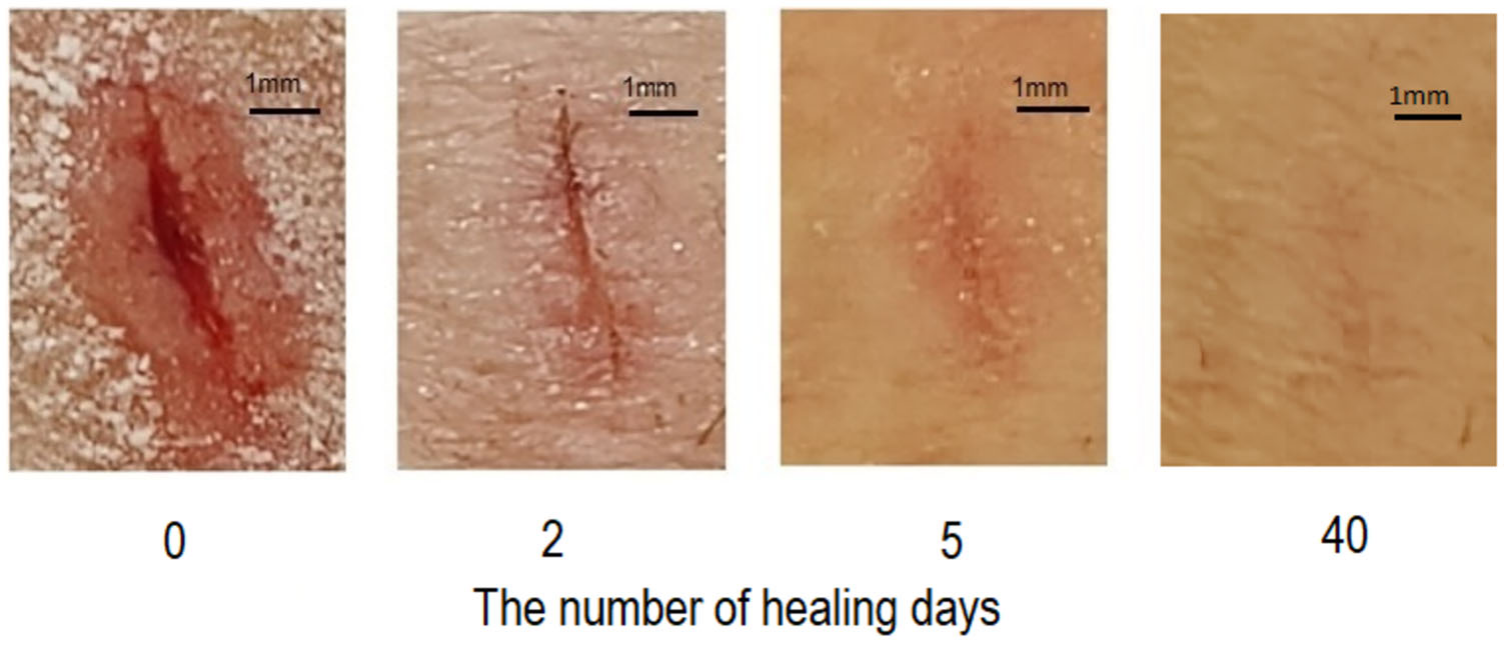
Visual observation of the degree of healing of wounds over time by treatment with a mixture in the form of a powder consisting of D-mannose and calcium propionate in a ratio of 40:1.

Figure 3 contains four images showing how wound healing progresses over time when wounds were treated with a powder mixture of D-mannose and calcium propionate in a ratio of 40:1. Each picture has a scale. The powder mixture of D-mannose with calcium propionate was applied to completely cover the wounds. The dressing did not adhere to the wound on any of the days. Of note was that on the wound treated with the powdered mixture of D-mannose with calcium propionate, the coagulated blood was mostly retained on the dressing for the first few days without the dressing sticking to the wound. It can be seen that the wound on the second day of this treatment has only a few traces of clotted blood. When the wound was treated only with D-mannose, it was observed that after the first 2 days, the wound was always completely covered with clotted blood. After 40 days, it can be seen that there are no visible traces of scars on the skin treated with the mixture of mannose and calcium propionate. This proves that this mixture can successfully prevent the appearance of scars and heal the wound in a short time.

## Discussion

Bleeding is the first stage of hemostasis. Calcium ions play a special role in hemostasis because they intervene in several stages of clot formation, being factors, and cofactors in the coagulation process [19]. In the human body, calcium is present in the form of salts with inorganic or organic acids, in the form of chelates by incorporation into proteins and in the form of free ionic calcium. Normal serum values of free calcium ion concentration are in the range of 1.1–1.3 mmol/L and total serum calcium in the range of 2.2–2.6 mmol/L with small differences depending on the analysis laboratory. Free ionic calcium has the most important physiological functions. The value is kept strictly within these limits by the actions of the calcium-sensing receptor, parathyroid hormone, and vitamin D.

We observed that bleeding increased in intensity when the wound was treated with a very high concentration of calcium ions obtained by treating the wound with calcium propionate powder alone. This increase in bleeding could be explained by calcium homeostasis, which through control systems tries to bring the concentration to nominal values by removing excess calcium ions from the wound by bleeding. In contrast, recent studies have shown that a slight increase in the concentration of calcium ions in plasma by the addition of calcium chloride induces platelet aggregation [20]. Based on these results, we studied how we can reduce the bleeding caused by calcium propionate powder by mixing it with D-mannose powder.

Figure 1 shows how the bleeding time is influenced by the increase in moles of D-mannose reported by one mole of calcium propionate. Both D-mannose and calcium propionate in powder form are highly soluble in water. By increasing the amount of D-mannose, a decrease in bleeding time was observed up to 20 moles of D-mannose per 1 mole of calcium propionate, after which the bleeding time remains relatively constant up to 60 moles of D-mannose and then the bleeding time increased to the value given by pure D-mannose. The decrease in bleeding time could be explained by the ability of calcium ions to form stable complexes with D-mannose, reducing practically the amount of free calcium ions in the blood of the wound to values that stimulate platelet aggregation. By increasing the amount of D-mannose, the amount of free calcium ions that will be retained in the D-mannose complex will also increase. This process will decrease the concentration of free calcium ions in the wound to values that influence platelet aggregation less and less. The complexation of free calcium ions is an equilibrium process, but among the alkaline earth metals, calcium forms the strongest complexes, while monovalent alkali metals such as sodium give complexes with low stability [21]. The nature of the anion does not appear to have any substantial effect. The α-D-mannose anomer gives more stable complexes because it has a higher degree of coordination, and therefore, in the presence of calcium ion there will be a substantial increase in this anomer. The complex formed is between a D-mannose molecule and a calcium ion [21]. The mixture of D-mannose powder with calcium propionate has the advantage that the bleeding time was shorter with approximately 50% compared to D-mannose when the mixture used had the molar ratio of D-mannose to calcium propionate in the range of 20 to 60.

From Fig. 2, it can be seen that when treating the wound with a mixture of D-mannose powder and calcium propionate powder in a ratio of 40:1, the healing time is reduced by approximately 40% compared to the treatment with D-mannose. The rapid healing of wounds is due to the synergistic action of D-mannose and calcium propionate. Both substances have the ability to protect the wound from external pathogens, but in different ways. In a recent study [13], it was shown that D-mannose is not an antiseptic, but it can immediately activate the innate immune system through mannose-binding lectin. Through this mechanism, a complex cascade process is started that will finally lead to the phagocytosis of a large number of pathogens through the intermediary of macrophages and neutrophils [13]. In addition, D-mannose influences the formation of thinner and denser fibrin fibers leading to a finer and stronger fibrin polymer [22]. This process implicitly leads to the deposition of less extracellular material and collagen in the wound matrix [23], which will be more rapidly removed from the wound. All of these lead to a reduction in healing time. In addition, the scars are no longer visible.

Calcium propionate is primarily known to combat some bacteria and fungi [14, 15]. Compared to D-mannose, propionate acts directly on pathogen metabolism, but the mode of action is not well known as it differs from pathogen to pathogen. Propionates have also been found to have anti-inflammatory properties [14]. Practically, when treating wounds with this mixture, the synergistic action of these two substances in the mixture can provide greater effectiveness and safety to the healing process because both substances contribute to the destruction of pathogens, as well as to the processes of new tissue formation.

Figure 3 shows a visual observation of the degree of healing of wounds over time by treatment with a mixture in the form of a powder consisting of D-mannose and calcium propionate in a ratio of 40:1. By this treatment, the extracellular matrix material that also contains red cells and forms scab over the wound is largely removed by retention on the dressing in the first days. In the treatment with D-mannose powder, the crust removal takes several days. This can be explained by the fact that calcium ions resulting from the dissociation of calcium propionate can also play an important role in accelerating wound healing through their action on the formation of granulation tissue, fibroblasts, and keratinocytes [19]. In this case, calcium ions play the role of extracellular regulator and secondary messengers in the proliferation and maturation processes of granular tissue, keratinocytes, and fibroblasts.

The results from this study may have some limitations, considering the relatively small number of male subjects, a small age range, and simple wounds. The first limitation was reduced because it was used a crossover experiment [24] and a homogeneous group of Caucasian men. Therefore, future studies should include a larger group of subjects, such as women and other age groups and abnormal scarring, or complicated wounds, so that there can be a more extensive validation of the results. In this study, the results obtained must be viewed with these limitations.

## Conclusion

This study found that bleeding time can be reduced to about 50% compared to D-mannose when treating the wound with a mixture containing 20-60 moles of D-mannose per 1 mole of calcium propionate. Our results show that wound healing time could be reduced by approximately 40% by treatment with a mixture of D-mannose and calcium propionate in powder form compared to treatment with D-mannose powder alone. No visible scarring was observed after 40 days on skin treated with this powdered mixture of D-mannose and calcium propionate.
